# Sonic Hedgehog Carried by Microparticles Corrects Angiotensin II-Induced Hypertension and Endothelial Dysfunction in Mice

**DOI:** 10.1371/journal.pone.0072861

**Published:** 2013-08-16

**Authors:** Vannina González Marrachelli, Maria Letizia Mastronardi, Mamadou Sarr, Raffaella Soleti, Daniela Leonetti, María Carmen Martínez, Ramaroson Andriantsitohaina

**Affiliations:** 1 LUNAM Université, Angers, France; 2 Institut National de la Sante et de la recherche Medicale U1063, Stress oxydant et pathologies métaboliques, Angers, France; 3 CHU Angers, Angers, France; Inserm, France

## Abstract

Microparticles are small fragments of the plasma membrane generated after cell stimulation. We recently showed that Sonic hedgehog (Shh) is present in microparticles generated from activated/apoptotic human T lymphocytes and corrects endothelial injury through nitric oxide (NO) release. This study investigates whether microparticles bearing Shh correct angiotensin II-induced hypertension and endothelial dysfunction in mice. Male Swiss mice were implanted with osmotic minipumps delivering angiotensin II (0.5 mg/kg/day) or NaCl (0.9%). Systolic blood pressure and heart rate were measured daily during 21 days. After 7 day of minipump implantation, mice received *i.v.* injections of microparticles (10 µg/ml) or *i.p*. Shh receptor antagonist cyclopamine (10 mg/kg/2 days) during one week. Angiotensin II induced a significant rise in systolic blood pressure without affecting heart rate. Microparticles reversed angiotensin II-induced hypertension, and cyclopamine prevented the effects of microparticles. Microparticles completely corrected the impairment of acetylcholine- and flow-induced relaxation in vessels from angiotensin II-infused mice. The improvement of endothelial function induced by microparticles was completely prevented by cyclopamine treatment. Moreover, microparticles alone did not modify NO and O_2_^. -^ production in aorta, but significantly increased NO and reduced O_2_^. -^ productions in aorta from angiotensin II-treated mice, and these effects were blocked by cyclopamine. Altogether, these results show that microparticles bearing Shh correct angiotensin II-induced hypertension and endothelial dysfunction in aorta through a mechanism associated with Shh-induced NO production and reduction of oxidative stress. These microparticles may represent a new therapeutic approach in cardiovascular diseases associated with decreased NO production.

## Introduction

Angiotensin II (Ang II), the principal effector peptide of the renin-angiotensin system, plays a major role in the initiation and progression of vascular diseases, such as hypertension, in part through reactive oxygen species [1]. Ang II-induced increase in reactive oxygen species in particular, superoxide (O_2_^·-^), leads to decreased bioavailability of nitric oxide (NO), which impairs endothelium-dependent vasodilatation and promotes vasoconstriction. Ang II-induced increase in blood pressure, vascular O_2_^·-^ levels, and endothelial dysfunction are improved either upon blockade of the system and/or the prevention of oxidative stress leading to an increase of NO bioavailability [2].

Microparticles (MPs) are small fragments generated from the plasma membrane after cell stimulation. The composition of MPs and the messages they transport (proteins, mRNA or miRNA) can differ depending on their origin [3]. MPs can be engineered to over-express different proteins by driving the synthesis of the relevant protein in MP-producing cells [4]. We have demonstrated that MPs released by apoptotic/stimulated human T lymphocytes harbor the morphogen Sonic hedgehog (Shh) and improve endothelial function in the mouse aorta by increasing NO release [5]. Also, endothelial dysfunction in mouse coronary artery after ischemia/reperfusion can be prevented by treatment with Shh-carrying MPs [5]. Moreover, MPs expressing Shh favor *in vitro* angiogenesis [6] and the recovery of hindlimb flow after peripheral ischemia through the activation of endothelial NO synthase and the increase of NO release and pro-angiogenic factor production [7]. The present study further aims to investigate whether MPs bearing Shh may correct Ang II-induced hypertension and endothelial dysfunction in mice.

## Materials and Methods

### MP production

The human lymphoid CEM T cell line (ATCC, Manassas, VA) was used for MP production. Cells were seeded at 10^6^ cells/ml and cultured in serum-free X-VIVO 15 medium (Cambrex, Walkersville, MD). MPs were produced as described previously [8]. Briefly, CEM cells were treated with phytohemagglutinin (5 µg/ml; Sigma-Aldrich, St. Louis, MO) for 72 h, then with phorbol-12-myristate-13 (20 ng/ml, Sigma-Aldrich) and actinomycin D (0.5 µg/ml, Sigma-Aldrich) for 24 h [8]. A supernatant was obtained by centrifugation at 750 *g* for 15 min, then at 1500 *g* for 5 min to remove cells and large debris, respectively. MPs from the supernatant were washed after three centrifugation steps (45 min at 14,000 *g*) and recovered in 400 µl NaCl (0.9% w/v). Washing medium for the last supernatant was used as control. Determination of the amount of MPs was carried out by measuring MP-associated proteins, using the method of Bradford, with BSA (Sigma-Aldrich) as the standard [5].

### Ethics statement

The procedure followed in the care and euthanasia of the study animals was in accordance with the Guide for the Care and Use of Laboratory animals published by US National Institutes of Health (NIH Publication No. 85-23, revised 1996) and was approved by the Ethical Committee for Animal Research of Angers University.

### Animals

Six groups of male Swiss mice (6-8 week old) were used: (i) group treated with infusion of saline by osmotic pump for 2 weeks, (ii) group receiving Ang II (Sigma-Aldrich, 0.5 mg/kg/day) infusion by osmotic pump for 2 weeks, (iii) group receiving saline by osmotic pump for 2 weeks and *i.v.* injection of MPs (10 µg/ml of blood) every two days over the last week, (iv) group receiving Ang II by osmotic pump for 2 weeks and *i.v.* injection of MPs every two days over the last week, (v) group receiving i.p. injection of cyclopamine (Biomol International, Plymouth Meeting, PA, 10 mg/kg) every two days over the last week, and (vi) group receiving Ang II infusion by osmotic pump for 2 weeks and *i.v.* injection of MPs every two days over the last week, and i.p. injection of cyclopamine. All experiments were conducted in mice housed in a temperature-controlled animal facility with a 12-hour light/dark cycle and free access to tap water and rodent chow.

### Ang II Infusion

Ang II at a dose of 0.5 mg/kg/day was delivered over 2 weeks via unprimed osmotic minipumps (Model 2004, Alzet Osmotic Pumps, Cupertino, CA) that were subcutaneously implanted into the back of mice. For control experiments mice were treated with saline delivered via osmotic minipumps. Animals were anesthetized with 2.5% isofluorane in 1.5 l/min O_2_ for the duration of the surgical implantation procedure. Buprenorphine (1mg/kg) in *s.c.* injection was administered immediately prior to surgery.

### Blood pressure measurements

Non-invasive blood pressure was measured by tail-cuff method (Letica, Barcelona, Spain). Briefly, all animals were trained everyday over a period of a week to get accustomed to the device. Measurements were performed prior to pump implantation over a week and 14 days after surgery. A total of 10 consecutive readings of systolic pressure and heart rate were daily recorded and averaged.

### Arterial preparations and mounting

Mice were euthanized via CO_2_ asphyxiation, and the thoracic aorta and the proximal segment of the small bowel were removed and pinned in a dissecting dish and cleaned of fat and connective tissue.

Segments of aorta (2 mm in length) were mounted on myographs filled with physiological salt solution (PSS). Aortic rings were stretched with a passive wall tension of 1 g. The PSS was continuously kept at 37°C and gassed with 95% O_2_ and 5% CO_2_ at pH 7.4. Isometric tension was recorded and collected by a force transducer. Cumulative acetylcholine (ACh, 1 nM -10 µM) concentration–response curves were obtained after pre-contraction of the artery with U46619 (80% of the maximal contractile response).

Branches II of mouse superior mesenteric arteries were mounted in arteriograph. Briefly, dissected arteries were mounted on two glass cannulas in the arteriograph chamber and attached with nylon ties. Arteries were bathed in PSS (pH 7.4; PO_2_ 160 mm Hg, PCO_2_ 37 mm Hg). Pressure was then set at 75 mm Hg. The presence of functional endothelium was assessed by the ability of ACh (10 µM) to induce more than 50% relaxation of vessels pre-contracted with U46619. To obtain active pressure versus diameter curves, diameter changes were measured at each step, when intraluminal pressure was increased from 10 to 125 mm Hg.

### NO and O_2_. ^-^ determination by electron paramagnetic resonance (EPR)

Aorta was incubated in a solution containing bovine serum albumin (20.5 g/l), CaCl_2_ (3mM), and L-arginine (0.8 mM) to assess NO production. A diethyldithiocarbamate-iron(II) complex (Fe[DETC]_2_) solution was added to the vessel and incubated for 45 min at 37°C. Then, aorta was immediately frozen using liquid nitrogen. Values are expressed in unit/mg weight of dried tissue. For O_2_^-^ detection, aorta was incubated in deferoxamine-chelated Krebs-Hepes solution containing 1-hydroxy-3-methoxycarbonyl-2,2,5,5-tetramethylpyrrolidine (CMH; 500 µM, Noxygen, Mainz, Germany), deferoxamine (25 µM, Sigma-Aldrich), and diethyldithiocarbamate (DETC, 5 µM, Sigma-Aldrich) at 37 °C for 20 min. NO and O_2_^-^ measurements were performed using a table-top x-band spectrometer Miniscope (Magnettech, MS200, Berlin, Germany). Recordings were made at 77 °K using a Dewar flask. The instrument setting was 10 mW of microwave power, 1 mT of amplitude modulation frequency, 60 s of sweep time and 3 scans. Values are expressed in unit/mg weight of dried aorta.

### Statistical Analysis

The results are expressed as means ± SEM. Comparisons among different groups were made by one-way ANOVA followed by Bonferroni *post hoc* test. *P* < 0.05 was considered to be statistically significant.

## Results

### Effects of MPs on systolic blood pressure and heart rate

**Figure 1:**
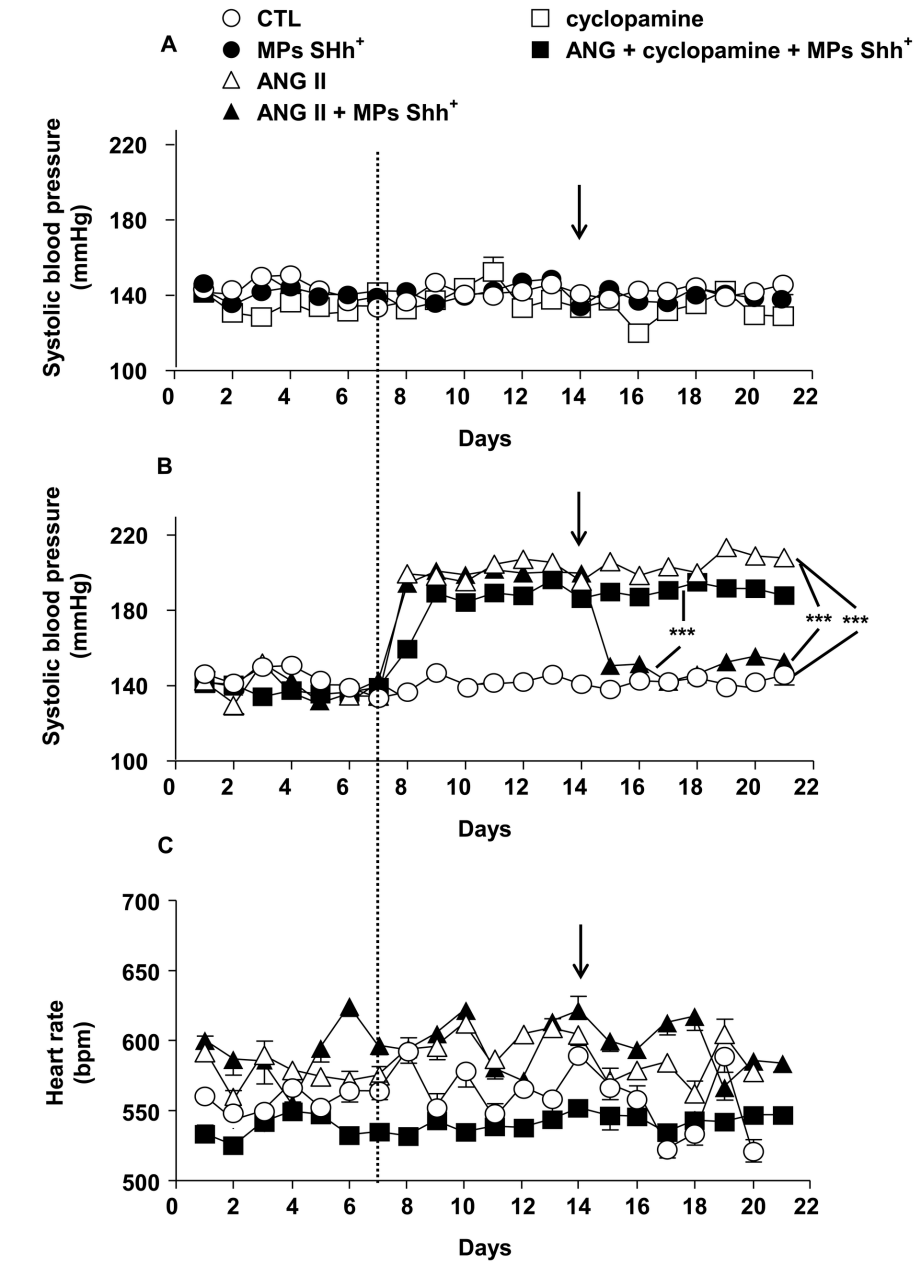
Time course of changes on systolic blood pressure and heart rate. Effects of either microparticles (MPs Shh+) or cyclopamine on systolic blood pressure (A). MPs Shh+ treatment abolished angiotensin II (Ang II)-induced hypertension. This effect was prevented by cyclopamine (B). Heart rate did not show differences between any groups (C). ***P<0.001. Results are given as means ± SE of 8-10 mice for each group. The variability of the responses is so small that the error bars cannot be observable with this size of symbols. The dashed line indicate the time at which the mice received the osmotic pump in the absence or presence of Ang II, the arrows indicate the time at which the mice received MPs Shh+ or vehicle by i.v. injection.

Systolic blood pressure was stable throughout the duration of the experimentation in control mice infused with saline and in those treated either with MPs alone or cyclopamine (Figure *1A*). Infusion of mice with Ang II resulted in a significant rise in blood pressure that was stable during its infusion (Figure *1B*). In another set of experiments when the hypertension induced by Ang II was stabilized, i.v. injection of MPs completely decreased systolic blood pressure towards the values of control animals. This effect of MPs lasted until the end of the experimental procedure. Interestingly, cyclopamine completely prevented the ability of MPs to restore the increase of blood pressure induced by Ang II infusion.

None of these treatments were associated with significant changes in heart rate values throughout the experiments (Figure 1C)

### MPs improve endothelial dysfunction induced by Ang II infusion

**Figure 2:**
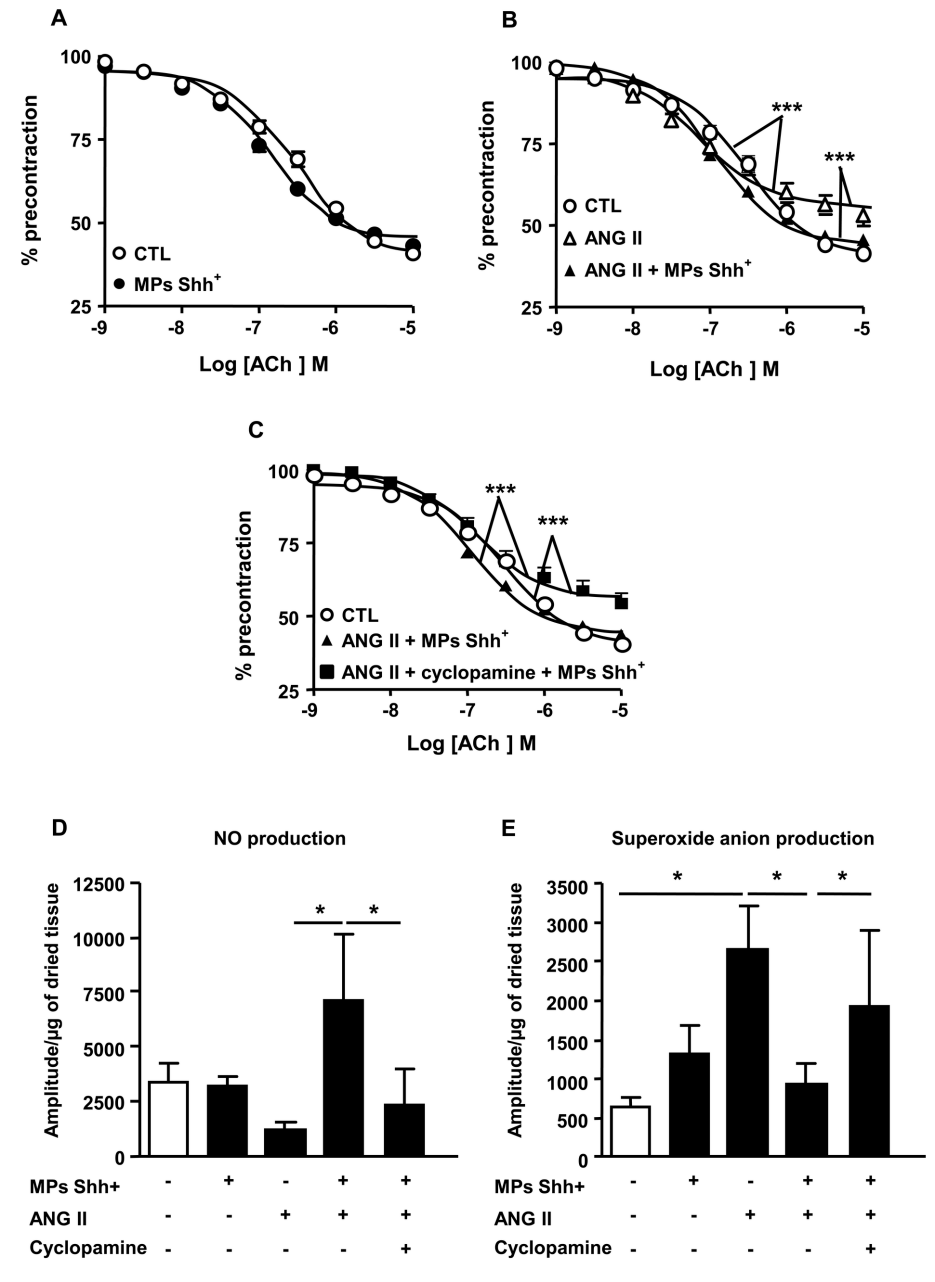
Concentration–response curves to acetylcholine (ACh) and nitric oxide and superoxide anion productions in aorta. Relaxation response curves to ACh (A–C) in aortic rings with endothelium precontracted with U-46619 from mice receiving saline (control, CTL), microparticles alone (MPs Shh+), angiotensin II (ANG II) alone, and the combination of ANG II plus MPs Shh+ in the absence and in the presence of cyclopamine. (D, E) Quantification of the amplitude of NO–Fe(DETC)_2_ (D) and O_2_-CMH (E) signals in aorta from mice receiving saline (CTL), MPs Shh+ alone, ANG II alone, and the combination of ANG II plus MPs Shh+ in the absence and in the presence of cyclopamine. Results are given as means ± SE of 8-15 mice for each group. *P<0.05, ***P<0.001.

The ACh-induced relaxation was not significantly different in aorta taken either from control or MP-treated mice (Figure *2A*). The endothelium-dependent relaxation to ACh was significantly impaired in aorta taken from mice injected with Ang II compared with those from mice injected with vehicle (Figure *2B*). The decrease in maximal response was not associated with changes of the sensitivity to the agonist. The endothelial dysfunction induced by Ang II treatment was entirely reversed after administration of MPs showing that MPs may preserve endothelial integrity and functionality in hypertension-induced endothelial injury (Figure *2B*). Interestingly, cyclopamine completely prevented the ability of MPs to correct endothelial dysfunction in vessels from Ang II-treated mice (Figure *2C*).

To evaluate whether MPs affect smooth muscle function, concentration–response curves to sodium nitroprusside were performed in aorta. The relaxation to sodium nitroprusside was not significantly different in vessels from the four groups of mice (not shown).

5-HT produced a concentration-dependent increase in tension in vessels of saline-treated animals with functional endothelium. MPs did not affect this response when used alone. As expected, infusion of angiotensin II induced hyperreactivity (Table *1*) which was not affected by MP treatment (Table *1*).

**Table 1:** Vascular responses to 5-HT of aortic rings of mice.

	**Aorta**	
	**pD_2_**	**E_max_**
**Control**	6.57 ± 0.02	2.15 ± 0.02
**+ MPs Shh+**	6.64 ± 0.02	2.18 ± 0.02
**+ Angiotensin II**	6.57 ± 0.008	2.90 ± 0.011***
**+ Angiotensin II + MPs Shh+**	6.48 ± 0.02	2.97 ± 0.03***
**+ Angiotensin II + cyclopamine + MPs Shh+**	6.22 ± 0.03*	2.78 ± 0.04*

Sensitivity (expressed as pD_2_) and maximal effect (E_max_) of aortic rings from control mice and from mice receiving microparticles (MPs Shh+) alone, angiotensin II alone, the combination of angiotensin II plus MPs Shh+ in the absence and in the presence of cyclopamine. Values are means ± SEM (8 mice for each group). *P<0.05, ***P<0.001 *versus* control.

### MPs prevent the decrease in NO production and the oxidative stress induced by Ang II

In aorta, MPs did modify neither NO nor O_2_^-^ production in comparison to saline-treated mice. By contrast, although the reduction of NO production in aorta taken from Ang II-treated mice was not significantly different, Ang II increased O_2_^-^ production (Figure 2D and 2E). Interestingly, MP treatment significantly enhanced NO production and reduced O_2_^-^ production in Ang II-treated mice. After blockade of the Shh pathway by cyclopamine, MP effects on NO production and oxidative stress were abolished (Figure 2D and 2E).

### Impaired flow-induced dilation by Ang II infusion is improved by MPs in small mesenteric arteries

**Figure 3:**
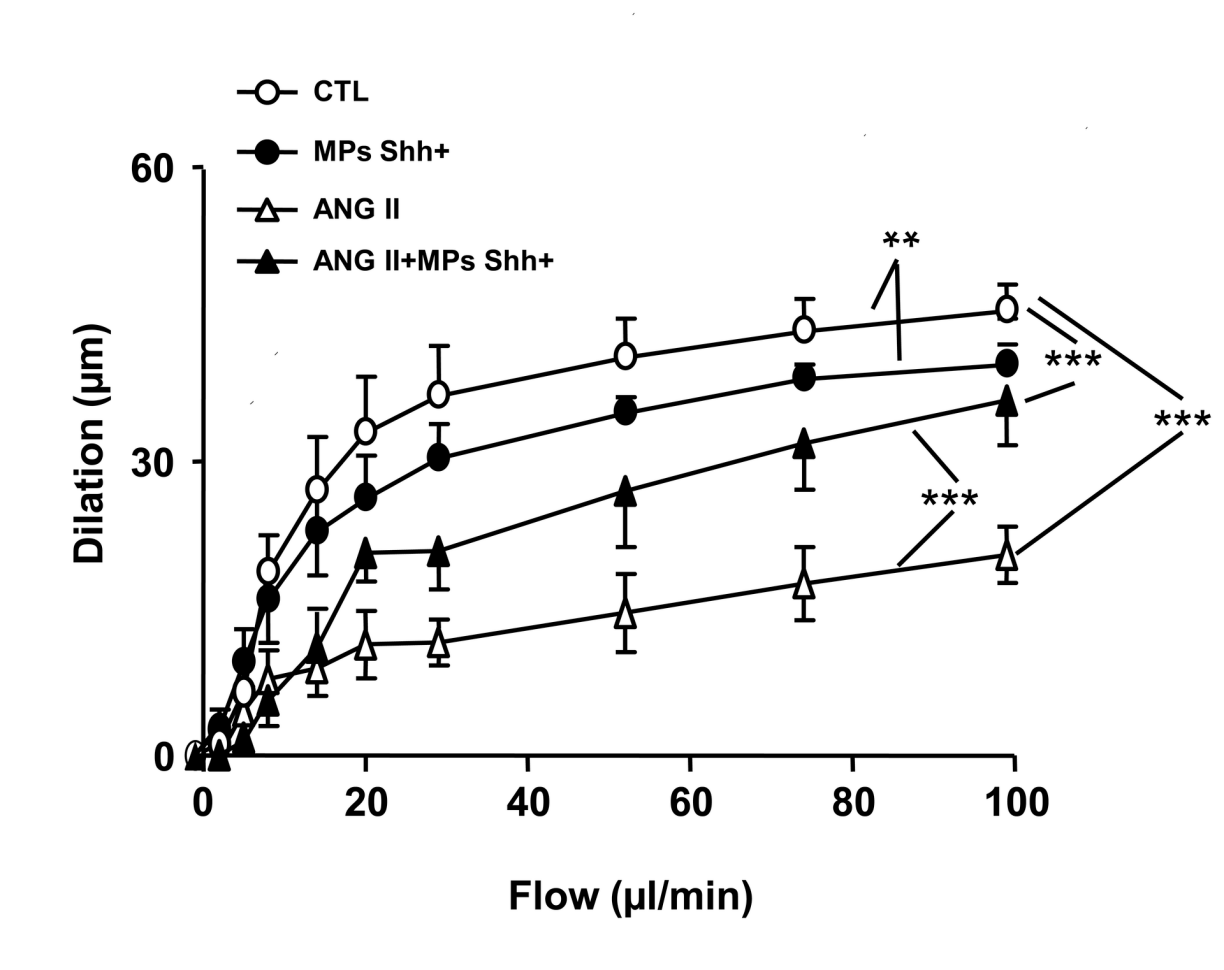
Vascular response of small mesenteric arteries to flow. Flow-induced dilation obtained in small mesenteric arteries from mice receiving saline (control, CTL), microparticles alone (MPs Shh+), angiotensin II (ANG II), and the combination of ANG II plus MPs Shh+. Results are given as means ± SE of 6-11 mice for each group. **P < 0.01 and ***P < 0.001.

In SMAs, Ang II infusion impaired flow-induced dilation when compared with vessels taken from saline treated mice (Figure *3*). MPs slightly reduced the flow-induced dilation, but it partially restored the attenuated dilation induced by Ang II-infusion (Figure *3*).

## Discussion

We report that MPs bearing Shh completely correct Ang II-induced hypertension without affecting heart rate via a pathway sensitive to Shh inhibitor. The beneficial effect of MPs was associated with the improvement of endothelial function of the response either to ACh or flow both in conductance and resistance arteries, respectively. However, MP treatment did not reverse the increase reactivity of the aorta to vasoconstrictor agent 5-HT. Of particular interest is that these effects of MPs were not due to change in sensitivity of the smooth muscle cells to NO but they were rather due to both increase of NO production and decrease of oxidative stress. Altogether, these results underscore the potent effect of MPs as an antihypertensive agent acting through an increase bioavailability of endothelial NO in conductance and resistance arteries.

Our strategy was to use MPs as pharmacological tools to reduce deleterious signaling in the vascular wall. For this purpose, the effect of MPs harboring Shh was assessed. This type of MPs improves endothelial function in the mouse aorta by increasing both eNOS expression and activity via PI3-kinase and Akt pathways and by reducing reactive oxygen species in human endothelial cells [5]. Also, endothelial dysfunction in mouse coronary artery after ischemia/reperfusion can be prevented by treatment with Shh-carrying MPs [5]. Moreover, MPs expressing Shh favor in vitro angiogenesis [6] and the recovery of hindlimb flow after peripheral ischemia through the activation of endothelial NO synthase and the increase of NO release and pro-angiogenic factor production [7]. Increased angiogenesis by MPs expressing Shh might participate in its ability to reduce vascular resistance and therefore vascular remodeling in Ang II-induced hypertension. However, in the present study, MPs expressing Shh^+^ slightly but significantly attenuated the ACh response in small mesenteric arteries (Figure 3) but did not affect ACh-response in the aorta under the same experimental conditions (Figure 2A). The differences between these results and those previously described [5] might be due to the duration of the treatment (one week *versus* 24 h, in the present study and in [5], respectively) and/or the vascular bed studied. In addition, MPs expressing Shh+ may harbor other proteins than Shh+, but also, mRNA and miRNA suggesting that the slightly attenuation of flow-induced dilation could not be induced by Shh+ but by other MP components. We cannot distinguish among these possibilities. Nevertheless, it is clearly shown that MPs Shh^+^ treatment restored endothelial dysfunction in the small mesenteric arteries in response to flow.

Recently, we have shown that MPs carrying Shh protect against apoptosis endothelial cells by a dual mechanism. On the one hand, MPs expressing Shh carry active antioxidant enzymes, catalase and isoforms of the superoxide dismutase, and on the other hand, they have the ability to increase the expression of manganese-superoxide dismutase in endothelial cells, through both internalization process and cyclopamine-sensitive mechanism [9]. All of these effects of MPs expressing Shh probably explain their ability to completely abrogate Ang II-induced hypertension and endothelial dysfunction in these mice. Indeed, the reduced vasodilation in response either to ACh or to flow in both conductance and resistance arteries was completely corrected upon MP treatment. Furthermore, these effects were associated with the ability of MPs to correct both the reduced NO production and the increased O_2_^. -^ in the vessel wall. It should be noted that both NO and O_2_^. -^ productions are variable but not the relaxation-induced by ACh. In this respect, in Ang II-induced hypertension, the relaxation to ACh involved other factors than NO, including reactive oxygen species from monoamine oxidases [10], NADPH oxidases and mitochondria, and cyclo-oxygenase-derived metabolites [11]. Thus, NO and O_2_^. -^ productions were not variable in aorta taken from control animals in which the other endothelial factors mentioned above are not produced. Thus, it is therefore not surprising to observe such apparent discrepancies. Nevertheless, the conclusion of the present manuscript still holds in as much at least the correction of endothelial function with respect to the changes in these two radicals participate in the antihypertensive effect of MPs Shh^+^.

Few studies have described the role of Shh pathway in hypertension. It has been shown that although Shh is upregulated in retinas exposed to ocular hypertension, and both exogenous and endogenous Shh have neuroprotective effects on damaged retinal ganglion cells, they did not affect intraocular pressure [12] Also in a model of obesity-associated hypertension, targeting adipocytes in mice fed a high-fat diet with human heme oxygenase-1 gene decreased adiposity and hypertension that was accompanied with increased Shh expression in adipocytes [13]. In the present study, since all effects of MPs were prevented by cyclopamine, one can advance the hypothesis that they act through a mechanism sensitive to blockade of Shh.

MP treatment was not however able to reverse the hyper-reactivity to 5-HT observed in Ang II-induced hypertensive animals. It is known that Ang II induces cyclo-oxygenase (COX)-2 expression and prostanoid production in vascular cell types such as endothelial cells, vascular smooth muscle cells, and adventitial fibroblasts as well as in whole vessels. Oxidative stress has been also suggested to induce COX activity or up-regulate COX-2 expression, and this is particularly increased in hypertension. Recently, an excess of reactive oxygen species from NADPH oxidase and/or mitochondria and the increased vascular COX-2/TP receptor axis act in concert to induce vascular dysfunction including increased vascular reactivity, and hypertension in the same experimental model [11]. Since MPs harboring Shh decrease oxidative stress [9] but are not able to counteract hyper-reactivity to 5-HT in aorta from Ang II- induced hypertensive mice, it is plausible to hypothesize that MPs are ineffective to affect the hyper-reactivity associated with COX-2/TP receptors activation. Further studies are needed to sort out the underlying mechanisms.

In conclusion, these findings suggest that Shh-positive MPs could represent a potent tool for stimulating NO release and reducing oxidative stress in the vessel wall to completely reverse Ang II-induced hypertension and extend the use of such MPs to treated disease states associated with endothelial dysfunction in addition to those associated with impaired angiogenesis.
